# Inflammatory fibroid polyp (Vanek's tumour), an unusual large polyp of the jejunum: a case report

**DOI:** 10.1186/1757-1626-2-7152

**Published:** 2009-05-18

**Authors:** Shafiq Rehman, Zakareya Gamie, Timothy R Wilson, Andrew Coup, Geetinder Kaur

**Affiliations:** 1 Department of Colorectal Surgery, Scunthorpe General HospitalCliff Gardens, Scunthorpe, North LincolnshireUnited Kingdom, DN15 7BH; 2 Department of Cellular Pathology, Lincoln County HospitalGreetwell Road, LincolnUnited Kingdom, LN2 5QY

## Abstract

We report the case of a 46-year-old man who presented with recurrent episodes of severe upper abdominal pain over a period of three months. A computerized tomography scan of his abdomen demonstrated a large non-obstructing jejunal mass. He underwent laparotomy and resection of a 13.5 cm tumour from the distal jejunum. Histopathological examination confirmed a large inflammatory fibroid polyp of the jejunum. The clinical presentation and microscopic features are discussed.

## Introduction

Inflammatory Fibroid Polyp (IFP) is an uncommon, benign, sub-mucosal lesion, first described by Vanek in 1949 [1]. When present, they are mostly found in the gastric antrum (70%) or in the ileum (20%); however, they are considered to be very rare in the duodenum and jejunum [2]. IFPs are usually found incidentally in asymptomatic patients [3]. However, patients can also present with abdominal pain, weight loss, bleeding, dyspeptic symptoms and obstruction, with the pattern of symptoms dependent on the site and size of the lesion [2]. We report a case of an IFP confined to the distal jejunum which presented in an unusual manner and review the literature.

## Case presentation

**Figure 1.**
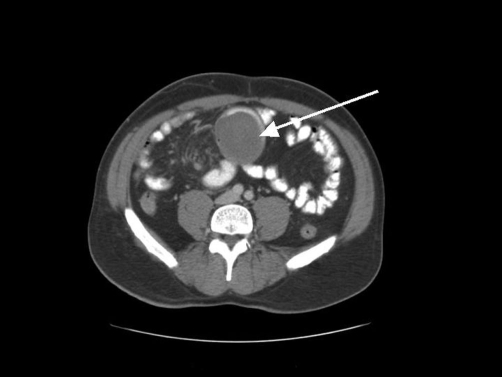
Enhanced CT scan of abdomen and pelvis demonstrating a well defined soft-tissue mass arising from the jejunum.

A previously well 46-year-old British Caucasian man was admitted with a 3 month history of a heavy dragging pain in his abdomen. The pain had been progressively worsening and was more noticeable when standing and moving. Despite having presented to medical personnel on a number of previous occasions, clinical examination revealed a 10-15 cm mobile mass in the centre of the abdomen. A plain abdominal radiograph did not show any features of obstruction. Ultrasonography of his abdomen prior to his admission had demonstrated gall bladder polyps, but no signs of cholelithiasis. Gastroscopy which had also been undertaken previously was found to be normal. A Computerized Tomography (CT) scan of his abdomen with oral and i.v. contrast was performed. This demonstrated a large soft tissue intraluminal mass arising from the distal jejunum measuring 11 cm × 8 cm × 5 cm with no signs of obstruction (Figure 1). The clinical impression was that of gastrointestinal stromal tumour (GIST), lymphoma or small bowel adenocarcinoma. A laparotomy with small bowel resection and side to side stapled anastomosis was performed and recovery was uneventful. Within hours of the operation, the patient reported that the heavy sensation in his abdomen had completely abated.

**Figure 2.**
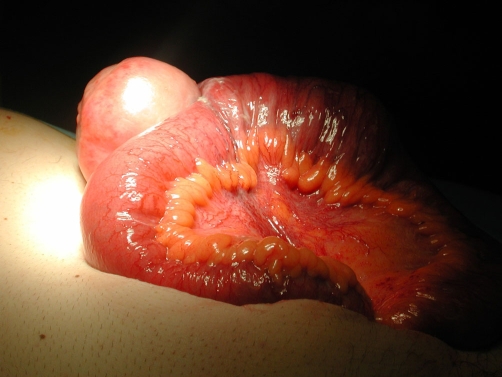
Specimen resected during the surgical procedure demonstrating a large jejunal IFP.

**Figure 3.**
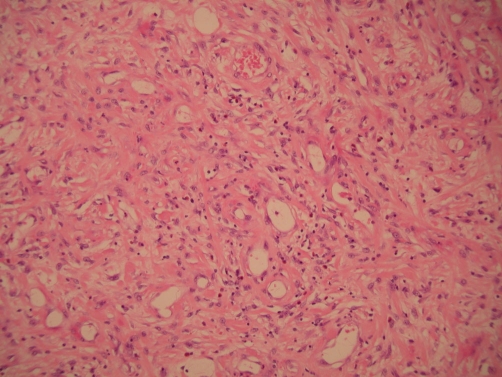
Histopathological examination of specimen revealed variable cellularity, and spindle cells having bland nuclei, and clear cytoplasm. There was an abundant inflammatory infiltrate comprising plasma cells, lymphocytes, and eosinophils.

Macroscopic examination of the specimen revealed a dumbbell-shaped, circumscribed fleshy white mass measuring 13.5 cm in maximum dimension (Figure 2). This extended into the mucosa, forming an ulcerated polyp with intact serosa. Histopathology revealed variable cellularity, with spindle cells having bland nuclei and clear cytoplasm. There was an abundant inflammatory infiltrate comprising plasma cells, lymphocytes and eosinophils (Figure 3). The lesion involved the entire thickness of the bowel wall with extensive ulceration of the overlying mucosa. On immunohistochemistry, the spindle cells were negative for cytokeratins, CD117 (c-kit), S100, Actin, Desmin, CD34, CD10 and Melan A. The morphological features were typical of IFP and the immunoprofile was consistent with this diagnosis. The patient recovered without complications and remained well and symptom free at 6 weeks post discharge.

## Discussion

IFPs are one of the least common benign small bowel tumours. They fall under the classification of submucosal connective tissue tumours and were first described by Vanek in 1949 [1]. They typically present in the 5th to 7th decade of life and can be found throughout the gastrointestinal (GI) tract but most commonly in the gastric antrum (70%) or ileum (20%), but rarely in the duodenum and jejunum [2]. Histologically they arise from the submucosa and are characterised by vascular and fibroblast proliferation and an inflammatory response, dominated by eosinophils [4]. Further immunohistochemical analysis can demonstrate variable reactivity for Actin, CD34, Desmin, CD117 and S100 [4]. The morphology is usually characteristic, but potential differential diagnoses of Vanek tumours on biopsy alone include GIST, inflammatory pseudotumour and other rare soft tissue lesions [5,6]. The aetiology of IFP is not known, but a mechanism of chemical, traumatic or metabolic mucosal injury with a poorly controlled inflammatory response has been hypothesised [7-9].

In the main, IFPs rarely reach more than 6 cm, and their size is presumably related to the likelihood of symptoms. Recently, Schildhaus *et al*., 2008 described 25 cases of small bowel IFPs, the largest of which measured 7 cm and none were found in the jejunum [10]. Recently a paper has described an IFP in the ileum of 15 cm [11]. Other recent papers have described ileal IFPs of smaller size [12,13] presenting with symptoms of obstruction. There have been reports of retroperitoneal IFPs measuring up to 20 cm [14] and of colon IFPs measuring upto 14 cm [15]. About 15 cases of jejunal IFP have been described in the current literature and these rarely exceed 3-4 cm [16].

Many IFPs are identified incidentally during endoscopy or laparotomy. When symptomatic, the clinical presentation relates to the site of the tumour. The symptoms are often vague and can include nausea, vomiting, dyspepsia, abdominal pain and change in bowel habit [3,16]. If the overlying mucosa ulcerates then GI bleeding or anaemia may occur. Acute presentations with intussusception or obstruction are also commonly described [17,18]. Most of the jejunal IFPs described in the literature to date have been found to cause small bowel obstruction as a result of intussusception. Surgical excision is the mainstay of treatment and the tumours are not thought to recur following complete resection [3].

In the current case report, the patient presented with a heavy pulling sensation in the abdomen. This was presumably due to the weight of the tumour pulling on the small bowel mesentery like a pendulum, which resolved immediately after surgery. To the best of our knowledge, this is the only reported case in which the tumour has presented in this manner. This could be explained by the unusually large size of the tumour involved (13.5 cm), which enlarged without causing obstruction or intussusception. The tumour in this case was easily palpable on abdominal examination. This case highlights the importance of performing a thorough examination, even when patients present with unusual symptoms that are suspicious of functional pain.

## List of abbreviations

IFP: Inflammatory Fibroid Polyp
GIST: Gastrointestinal stromal tumour
CT: Computerized Tomography
GI: Gastrointestinal
